# Supplementary material for: Exposure to Non-Steady-State Oxygen Is Reflected in Changes to Arterial Blood Gas Values, Prefrontal Cortical Activity, and Systemic Cytokine Levels
Source: Int J Mol Sci. 2024 Mar 14;25(6):3279. doi: 10.3390/ijms25063279 (PMC10969887; doi:10.3390/ijms25063279)
Supplement: Supplementary file 1 [file ijms-25-03279-s001.zip › ijms-2878485-supplementary.pdf]

Supplemental Table S1: Arterial Blood P_aCO_2 Comparisons. Supplemental Table 1 presents arterial blood sample P_aCO_2 values measured immediately upon reaching the predetermined F_iO_2 level (following the final non-steady-state exposure) with those measured 120 seconds later during steady-state F_iO_2. No significant differences were found. Comparisons were performed using paired *t*-tests unless otherwise indicated. †Sample size decreased due to one participant whose arterial line catheter became non-functional during the latter part of the experimental protocol. **p*-value from Wilcoxon Signed Rank test. Adj. *p*-values from false discovery rate (FDR).

	**F_iO_2 range during non-steady-state/steady-state hyperoxia exposure**	**N†**	**P_aCO_2 Measured upon reaching predetermined F_iO_2 level following non-steady-state/steady-state hyperoxia exposure** (Mean ± SD) *Range*	**P_aCO_2 Measured after maintaining predetermined F_iO_2 level for 120 seconds** (Mean ± SD) *Range*	***p*-value**	**Adj. *p*-value**
**749 mmHg**	21% steady-state	24		38.87 ± 3.40 *(30.80-44.30)*	n/a	
	35% ± 15%	24	41.13 ± 4.74 *(30.60-49.20)*	40.48 ±4.46 *(29.60-46.70)*	0.1898	0.5694
	50% ± 15%	24	41.79 ± 4.52 *(30.60-48.70)*	41.64 ±4.55 *(28.60-51.90)*	0.7091	0.9591
	65% ± 15%	24	41.81 ± 4.21 *(30.40-48.40)*	42.53 ± 3.91 *(32.10-50.00)*	0.1235	0.5694
	80% ± 15%	24	43.56 ±3.28 *(36.90-48.10)*	42.83 ± 3.70 *(32.90-50.10)*	0.1790	0.5694
	100% steady-state	24		44.02 ± 4.43 *(34.80-50.80)*	n/a	
**565 mmHg**	21% steady-state	24		34.97 ± 3.91 *(25.40-42.40)*	n/a	
	35% ± 15%	24	39.80 ± 4.41 *(25.80-46.70)*	39.83 ± 4.53 *(24.30-46.90)*	0.9649	0.9649
	50% ± 15%	24	39.68 ± 4.38 *(26.80-45.20)*	40.57 ± 4.66 *(27.10-49.60)*	0.1151	0.5694
	65% ± 15%	23	41.38 ± 3.93 *(31.10-48.20)*	41.80 ± 5.04 *(28.10-56.30)*	0.5261	0.9591
	80% ± 15%	23	42.00 ± 4.62 *(28.90-49.00)*	42.08 ± 4.24 *(30.00-48.00)*	0.8475	0.9591
	100% steady-state	23		42.96 ± 4.17 *(33.40-50.00)*	n/a	
**494 mmHg**	21% steady-state	23		33.15 ± 2.97 *(29.20-38.50)*	n/a	
	35% ± 15%	23	38.10 ± 5.01 *(28.90-46.90)*	37.89 ± 4.30 *(29.00-44.80)*	0.7007	0.9591
	50% ± 15%	23	39.25 ± 4.48 *(31.10-47.80)*	39.33± 4.52 *(28.70-47.20)*	0.8792	0.9591
	65% ± 15%	23	39.90 ± 4.79 *(27.20-45.50)*	40.02 ± 5.36 *(25.30-48.20)*	0.8418	0.9591
	80% ± 15%	23	39.90 ± 5.36 *(24.00-49.30)*	40.18 ± 5.23 *(26.30-47.60)*	0.6383	0.9591
	100% steady-state	23		41.01 ± 4.89 *(26.10-49.00)*	n/a	

Supplemental Table S2: Arterial Blood pH Comparisons. Supplemental Table 2 presents arterial blood sample pH values measured immediately upon reaching the predetermined F_iO_2 level (following

the final non-steady-state exposure) with those measured 120 seconds later during steady-state F_iO_2. Comparisons were performed using paired *t*-tests unless otherwise indicated. †Sample size decreased due to one participant whose arterial line catheter became non-functional during the latter part of the experimental protocol. *p-value from Wilcoxon Signed Rank test; Adj. p-values from false discovery rate (FDR).

	**F_iO_2 range during non-steady-state/steady-state hyperoxia exposure**	**N†**	**pH Measured upon reaching predetermined F_iO_2 level following non-steady-state/steady-state hyperoxia exposure** (Mean ± SD) *Range*	**pH Measured after maintaining predetermined F_iO_2 level for 120 seconds** (Mean ± SD) *Range*	***p*-value**	**Adj. *p*-value**
**749 mmHg**	21% steady-state	24		7.40 ± 0.02 *(7.36-7.45)*	n/a	
	35% ± 15%	24	7.40 ± 0.03 *(7.36-7.46)*	7.40 ± 0.03 *(7.35-7.50)*	0.2069	0.6207
	50% ± 15%	24	7.40 ± 0.03 *(7.36-7.47)*	7.40 ± 0.03 *(7.34-7.50)*	0.3845	0.9048
	65% ± 15%	24	7.41 ± 0.03 *(7.35-7.47)*	7.41 ± 0.03 *(7.36-7.48)*	0.9817	0.9817
	80% ± 15%	24	7.40 ± 0.02 *(7.37-7.44)*	7.41 ± 0.03 *(7.36-7.49)*	0.0264*	0.1584
	100% steady-state	24		7.41 ± 0.03 *(7.36-7.47)*	n/a	
**565 mmHg**	21% steady-state	24		7.43 ± 0.03 *(7.39-7.52)*	n/a	
	35% ± 15%	24	7.40 ± 0.03 *(7.35-7.50)*	7.40 ± 0.03 *(7.37-7.54)*	0.0247*	0.1584
	50% ± 15%	24	7.41 ± 0.04 *(7.35-7.50)*	7.40 ± 0.03 *(7.35-7.49)*	0.0798	0.3192
	65% ± 15%	23	7.40 ± 0.02 *(7.36-7.46)*	7.40 ± 0.03 *(7.37-7.49)*	0.7338	0.9114
	80% ± 15%	23	7.40 ± 0.03 *(7.36-7.48)*	7.40 ± 0.03 *(7.37-7.47)*	0.5769	0.9114
	100% steady-state	23		7.40 ± 0.03 *(7.36-7.45)*	n/a	
**494 mmHg**	21% steady-state	23		7.45 ± 0.02 *(7.41-7.48)*	n/a	
	35% ± 15%	23	7.41 ± 0.03 *(7.36-7.48)*	7.41 ± 0.03 *(7.37-7.46)*	0.7511	0.9114
	50% ± 15%	23	7.40 ± 0.03 *(7.35-7.47)*	7.40 ± 0.03 *(7.36-7.51)*	0.7595	0.9114
	65% ± 15%	23	7.40 ± 0.04 *(7.35-7.51)*	7.40 ± 0.04 *(7.36-7.55)*	0.9494	0.9817
	80% ± 15%	23	7.41 ± 0.05 *(7.36-7.57)*	7.41 ± 0.04 *(7.36-7.55)*	0.4524	0.9048
	100% steady-state	23		7.41 ± 0.04 *(7.36-7.55)*	n/a	

Supplemental Table S3: Arterial Blood HCO_3^- Comparisons. Supplemental Table 3 presents arterial blood sample HCO_3 values measured immediately upon reaching the predetermined F_iO_2 level (following the final non-

steady-state exposure) with those measured 120 seconds later during steady-state F_iO_2. Comparisons were performed using paired *t*-tests unless otherwise indicated. †Sample size decreased due to one participant whose arterial line catheter became non-functional during the latter part of the experimental protocol.
*p-value from Wilcoxon Signed Rank test; Adj. *p*-values from false discovery rate (FDR).

	**F_iO_2 range during non-steady-state/steady-state hyperoxia exposure**	**N†**	**HCO_3 Measured upon reaching predetermined F_iO_2 level following non-steady-state/steady-state hyperoxia exposure** (Mean ± SD) *Range*	**HCO_3 Measured after maintaining predetermined F_iO_2 level for 120 seconds** (Mean ± SD) *Range*	***p*-value**	**Adj. *p*-value**
**749 mmHg**	21% steady-state	24		24.15 ± 1.65 *(20.50-27.00)*	n/a	
	35% ± 15%	24	25.27 ± 1.94 *(21.10-27.70)*	25.16 ± 1.70 *(20.60-27.50)*	0.5871	0.8614
	50% ± 15%	24	25.82 ± 1.81 *(21.40-29.20)*	25.92 ± 1.75 *(22.30-29.00)*	0.6235	0.8614
	65% ± 15%	24	26.19 ± 1.58 *(22.30-28.60)*	26.63 ± 1.45 *(23.80-29.70)*	0.0310*	0.1860
	80% ± 15%	24	27.11 ± 1.46 *(23.80-29.80)*	27.04 ± 1.42 *(24.60-29.80)*	0.7315	0.8614
	100% steady-state	24		27.67 ± 1.78 *(24.50-31.50)*	n/a	
**565 mmHg**	21% steady-state	24		23.28 ± 1.49 *(20.50-25.90)*	n/a	
	35% ± 15%	24	24.41 ± 1.52 *(20.00-27.80)*	24.85 ± 1.72 *(20.60-28.40)*	0.0284*	0.1860
	50% ± 15%	24	24.98 ± 1.64 *(20.91-27.40)*	25.05 ± 1.69 *(20.80-28.20)*	0.6969	0.8614
	65% ± 15%	23	25.77 ± 1.73 *(22.10-29.00)*	25.91 ± 2.12 *(21.60-32.40)*	0.7896*	0.8614
	80% ± 15%	23	26.04 ± 1.63 *(21.70-28.90)*	26.23 ± 1.78 *(21.50-29.80)*	0.2933	0.8614
	100% steady-state	23		26.57 ± 1.60 *(23.40-30.30)*	n/a	
**494 mmHg**	21% steady-state	23		22.88 ± 1.47 *(19.80-26.80)*	n/a	
	35% ± 15%	23	24.01 ± 1.76 *(20.20-27.40)*	23.82 ± 1.59 *(20.60-27.20)*	0.2407	0.8614
	50% ± 15%	23	24.29 ± 1.34 *(21.80-26.70)*	24.41 ± 1.64 *(21.90-27.90)*	0.5067	0.8614
	65% ± 15%	23	24.83 ± 1.50 *(21.70-27.40)*	24.90 ± 1.73 *(21.90-28.00)*	0.6581	0.8614
	80% ± 15%	23	25.19 ± 1.73 *(21.70-28.80)*	25.19 ± 1.79 *(22.10-28.60)*	0.9858	0.9858
	100% steady-state	23		25.56 ± 1.57 *(22.80-28.10)*	n/a	

Supplemental Table S4: Arterial Blood SaO_2 Comparisons. Supplemental Table 4 presents arterial blood sample SaO_2 values measured immediately upon reaching the predetermined F_iO_2 level (following the final non-steady-state exposure) with those measured 120 seconds later during steady-state F_iO_2. Comparisons were performed using paired *t*-tests unless otherwise indicated. †Sample size decreased due to one participant whose arterial line catheter became non-functional during the latter part of the experimental protocol. **p*-value from Wilcoxon Signed Rank test; Adj. *p*-values from false discovery rate (FDR).

	**F_iO_2 range during non-steady-state/steady-state hyperoxia exposure**	**N†**	**SaO_2 Measured upon reaching predetermined F_iO_2 level following non-steady-state/steady-state hyperoxia exposure** (Mean ± SD) *Range*	**SaO_2 Measured after maintaining predetermined F_iO_2 level for 120 seconds** (Mean ± SD) *Range*	***p*-value***	**Adj. *p*-value**
**749 mmHg**	21% steady-state	24		97.29 ± 1.12 *(94.00-99.00)*	n/a	
	35% ± 15%	24	99.96 ± 0.20 *(99.00-100.00)*	99.71 ± 0.69 *(97.00-100.00)*	0.0625	0.0938
	50% ± 15%	24	99.96 ± 0.20 *(99.00-100.00)*	99.96 ± 0.20 *(99.00-100.00)*	---	
	65% ± 15%	24	100.00 ± 0.00 *(100.00-100.00)*	100.00 ± 0.00 *(100.00-100.00)*	---	
	80% ± 15%	24	100.00 ± 0.00 *(100.00-100.00)*	100.00 ± 0.00 *(100.00-100.00)*	----	
	100% steady-state	24		100.00 ± 0.00 *(100.00-100.00)*	n/a	
**565 mmHg**	21% steady-state	24		93.38 ± 2.18 *(86.00-97.00)*	n/a	
	35% ± 15%	24	99.21 ± 0.51 *(98.00-100.00)*	98.63 ± 0.77 *(63.00-100.00)*	0.0002*	0.0006
	50% ± 15%	24	99.92 ± 0.28 *(99.00-100.00)*	99.54 ± 1.06 *(95.00-100.00)*	0.0313*	0.0563
	65% ± 15%	23	100.00 ± 0.00 *(100.00-100.00)*	99.96 ± 0.21 *(99.00-100.00)*	1.0000	1.0000
	80% ± 15%	23	100.00 ± 0.00 *(100.00-100.00)*	100.96 ± 0.21 *(99.00-100.00)*	1.0000	1.0000
	100% steady-state	23		100.00 ± 0.00 *(100.00-100.00)*	n/a	
**494 mmHg**	21% steady-state	23		84.42 ± 4.34 *(73.00-92.00)*	n/a	
	35% ± 15%	23	97.96 ± 1.00 *(95.00-99.00)*	95.04 ± 2.55 *(87.00-98.00)*	<.0001*	0.0005
	50% ± 15%	23	99.26 ± 0.69 *(97.00-100.00)*	98.65 ± 0.78 *(96.00-99.00)*	0.0002*	0.0006
	65% ± 15%	23	99.87 ± 0.46 *(98.00-100.00)*	99.61 ± 0.72 *(97.00-100.00)*	0.0313*	0.0563
	80% ± 15%	23	100.00 ± 0.00 *(100.00-100.00)*	99.91 ± 0.42 *(98.00-100.00)*	1.0000	1.0000
	100% steady-state	23		100.00 ± 0.00 *(100.00-100.00)*	n/a	

Supplemental Table S5: Arterial Blood Glucose Comparisons. Supplemental Table 5 presents arterial blood sample glucose values measured immediately upon reaching the predetermined F_iO_2 level (following the final non-steady-state exposure) with those measured 120 seconds later during steady-state F_iO_2. No significant differences were found. Comparisons were performed using paired *t*-tests unless otherwise indicated. †Sample size decreased due to one participant whose arterial line catheter became non-functional during the latter part of the experimental protocol. **p*-value from Wilcoxon Signed Rank test. Adj. *p*-values from false discovery rate (FDR).

	**F_iO_2 range during non-steady-state/steady-state hyperoxia exposure**	**N†**	**Glucose Measured upon reaching predetermined F_iO_2 level following non-steady-state/steady-state hyperoxia exposure** (Mean ± SD) *Range*	**Glucose Measured after maintaining predetermined F_iO_2 level for 120 seconds** (Mean ± SD) *Range*	***p*-value**	**Adj. *p*-value**
**749 mmHg**	21% steady-state	24		100.83 ± 11.36 *(84.00-129.00)*	n/a	
	35% ± 15%	24	104.08 ± 14.33 *(87.00-148.00)*	103.13 ± 13.34 *(87.00-142.00)*	0.1383	0.4149
	50% ± 15%	24	103.25 ± 14.18 *(87.00-143.00)*	103.71 ± 14.21 *(84.00-143.00)*	0.5156	0.6997
	65% ± 15%	24	102.25 ± 13.34 *(85.00-138.00)*	102.75 ± 12.86 *(86.00-137.00)*	0.3537	0.6063
	80% ± 15%	24	102.67 ± 12.27 *(85.00-135.00)*	101.96 ± 11.82 *(84.00-133.00)*	0.0985	0.4149
	100% steady-state	24		102.21 ± 11.22 *(85.00-133.00)*	n/a	
**565 mmHg**	21% steady-state	24		100.08 ± 10.76 *(83.00-128.00)*	n/a	
	35% ± 15%	24	99.96 ± 10.58 *(85.00-119.00)*	100.83 ± 10.81 *(84.00-121.00)*	0.0673	0.4149
	50% ± 15%	24	101.71 ± 11.57 *(81.00-132.00)*	101.50 ± 11.08 *(85.00-128.00)*	0.6026	0.7231
	65% ± 15%	23	100.48 ± 11.73 *(83.00-131.00)*	100.43 ± 11.58 *(84.00-127.00)*	0.8981	0.8981
	80% ± 15%	23	100.30 ± 10.83 *(82.00-127.00)*	100.61 ± 10.16 *(83.00-127.00)*	0.3282	0.6063
	100% steady-state	23		99.75 ± 9.97 *(80.00-124.00)*	n/a	
**494 mmHg**	21% steady-state	23		97.79 ± 8.85 *(82.00-115.00)*	n/a	
	35% ± 15%	23	96.78 ± 8.98 *(82.00-118.00)*	97.30 ± 9.33 *(80.00-117.00)*	0.1739	0.4174
	50% ± 15%	23	99.13 ± 8.42 *(84.00-117.00)*	99.70 ± 8.57 *(85.00-119.00)*	0.1196	0.4149
	65% ± 15%	23	99.87 ± 8.55 *(85.00-119.00)*	99.83 ± 8.51 *(84.00-121.00)*	0.8701	0.8981
	80% ± 15%	23	98.87 ± 8.65 *(85.00-124.00)*	99.13 ± 8.01 *(82.00-119.00)*	0.5248	0.6997
	100% steady-state	23		98.78 ± 8.53 *(83.00-121.00)*	n/a	

Supplemental Table S6: Arterial Blood Hematocrit Comparisons. Supplemental Table 6 presents arterial blood sample hematocrit values measured immediately upon reaching the predetermined F_iO_2 level (following the final non-steady-state exposure) with those measured 120 seconds later during steady-state F_iO_2. Comparisons were performed using paired *t*-tests unless otherwise indicated. †Sample size decreased due to one participant whose arterial line catheter became non-functional during the latter part of the experimental protocol. **p*-value from Wilcoxon Signed Rank test; Adj. *p*-values from false discovery rate (FDR).

	**F_iO_2 range during non-steady-state/steady-state hyperoxia exposure**	**N†**	**Hematocrit Measured upon reaching predetermined F_iO_2 level following non-steady-state/steady-state hyperoxia exposure** (Mean ± SD) *Range*	**Hematocrit Measured after maintaining predetermined F_iO_2 level for 120 seconds** (Mean ± SD) *Range*	***p*-value**	**Adj. *p*-value**
**749 mmHg**	21% steady-state	24		41.54 ± 2.98 *(35.00-46.00)*	n/a	
	35% ± 15%	24	41.71 ± 3.13 *(35.00-46.00)*	41.54 ± 3.04 *(35.00-46.00)*	0.5596	0.6451
	50% ± 15%	24	41.13 ± 3.42 *(34.00-46.00)*	41.88 ± 3.04 *(35.00-47.00)*	0.0098*	0.1176
	65% ± 15%	24	41.46 ± 3.32 *(34.00-47.00)*	41.75 ± 3.07 *(35.00-46.00)*	0.1431	0.5724
	80% ± 15%	24	41.75 ± 2.95 *(35.00-46.00)*	41.58 ± 3.16 *(35.00-46.00)*	0.2891	0.6451
	100% steady-state	24		41.50 ± 3.18 *(34.00-46.00)*	n/a	
**565 mmHg**	21% steady-state	24		41.88 ± 3.21 *(35.00-47.00)*	n/a	
	35% ± 15%	24	42.00 ± 3.23 *(35.00-47.00)*	42.08 ± 3.08 *(36.00-47.00)*	0.5913	0.6451
	50% ± 15%	24	41.67 ± 3.14 *(35.00-46.00)*	42.00 ± 3.01 *(35.00-46.00)*	0.0781	0.4686
	65% ± 15%	23	41.87 ± 3.20 *(34.00-47.00)*	42.00 ± 3.15 *(35.00-47.00)*	0.4551	0.6451
	80% ± 15%	23	41.78 ± 3.01 *(35.00-46.00)*	41.91 ± 3.23 *(34.00-47.00)*	0.5625	0.6451
	100% steady-state	23		42.08 ± 3.27 *(35.00-47.00)*	n/a	
**494 mmHg**	21% steady-state	23		42.08 ± 3.20 *(35.00-47.00)*	n/a	
	35% ± 15%	23	42.13 ± 3.24 *(35.00-47.00)*	42.30 ± 3.31 *(35.00-47.00)*	0.3438	0.6451
	50% ± 15%	23	42.04 ± 3.48 *(34.00-47.00)*	42.09 ± 3.38 *(34.00-47.00)*	1.0000	1.0000
	65% ± 15%	23	42.09 ± 3.42 *(34.00-48.00)*	42.26 ± 3.12 *(35.00-47.00)*	0.3984	0.6451
	80% ± 15%	23	41.91 ± 3.41 *(34.00-47.00)*	42.13 ± 3.28 *(35.00-47.00)*	0.3398	0.6451
	100% steady-state	23		42.22 ± 3.26 *(35.00-47.00)*	n/a	
